# Supplementary material for: Evaluation of the Bangkok Health Research and Ethics Interest Group: reflecting on the experiences of group members, researchers and facilitators participating in an urban community advisory board in Thailand
Source: Res Involv Engagem. 2026 Mar 5;12:30. doi: 10.1186/s40900-026-00860-1 (PMC12961771; doi:10.1186/s40900-026-00860-1)
Supplement: Supplementary file 1 — Supplementary Material 1 [file 40900_2026_860_MOESM1_ESM.docx]

# Evaluation of the Bangkok Health Research and Ethics Interest Group (HREIG): reflecting on the experiences of group members, researchers and facilitators participating in an urban community advisory board in Thailand

**Anne Osterrieder* ^1, 2^, Supanat Ruangkajorn^1^, Bhensri Naemiratch^1^, Tassawan Poomchaichote^1^, Supa-at Asarath^1^, Kanpong Boonthaworn^1^, Phaik Yeong Cheah^1, 2^**

**Affiliations**

^1^ Mahidol-Oxford Tropical Medicine Research Unit, Faculty of Tropical Medicine, Mahidol University, Bangkok, Thailand.

^2^ Centre for Tropical Medicine & Global Health, Nuffield Department of Medicine, University of Oxford, Oxford, UK.

* Corresponding author: Anne Osterrieder ([anne.osterrieder@ndm.ox.ac.uk](mailto:anne.osterrieder@ndm.ox.ac.uk))

**Supplementary Figure 1:** Diagram showing the HREIG logic model, which was used as tool in the HREIG evaluation study. A logic model is a visual map/diagram of the pathways that lead from the project activities and inputs to anticipated project outputs and outcomes.

*Based on the ‘Logic Model Development Guide’ by the W.K. Kellog Foundation (2004)*
